# Associations between neonatal serum bilirubin and childhood hypertension

**DOI:** 10.1371/journal.pone.0219942

**Published:** 2019-07-18

**Authors:** Huan Yu, Lile Zou, Yuan He, Lijuan Luo, Wenbin Dong, Yongjun Zhang, Xiaoping Lei

**Affiliations:** 1 Department of Neonatology, Affiliated Hospital of Southwest Medical University, Luzhou, Sichuan, China; 2 Department of Histology and Embryology, Southwest Medical University, Luzhou, Sichuan, China; 3 Department of Neonatology, Xinhua Hospital, Shanghai Jiao Tong University School of Medicine, Shanghai, China; 4 Birth Defects Clinical Medical Research Center of Sichuan Province, Luzhou, Sichuan, China; University of Hong Kong, CHINA

## Abstract

Mild hyperbilirubinemia is inversely associated with cardiometabolic diseases in adults. The aim of this study was to evaluate the association between neonatal serum bilirubin levels and childhood hypertension. Data were obtained from the U.S. Collaborative Perinatal Project conducted at 12 U.S. medical centers from 1959 to 1965. This multicenter study recruited participants before phototherapy was routinely used, thereby excluding the influence of phototherapy. In 37,544 newborns (31,819 term and 5,725 preterm births), a generalized linear model and a logistic regression model were used to calculate the linear coefficients and adjusted odds ratios (ORs) of blood pressure and hypertension at 7 years of age based on neonatal serum bilirubin levels. No significant correlation was observed between serum bilirubin at 48 hours after birth and blood pressure at the age of 7 years in the whole study population and in the subgroup of term infants. In preterm infants, a lower total serum bilirubin and unconjugated bilirubin of 3 mg/dl were associated with a higher systolic blood pressure of 62 mmHg (0.38–0.86, p <0.001) and 0.70 mmHg (0.10–1.30, p <0.05) respectively. Relative to a total serum bilirubin level <3 mg/dl among preterm infants, total serum bilirubin levels of 3–6 mg/dl (adjusted OR 1.36; 95% CI: 0.98–1.89), 6–9 mg/dl (adjusted OR 1.35; 95% CI: 0.98–1.85), 9–12 mg/dl (adjusted OR 1.55; 95% CI: 1.10–2.19), and ≥12 mg/dl (adjusted OR 1.42; 95% CI: 1.01–2.00) were associated with higher risks of hypertension. After stratifying for the subtypes of bilirubin, the associations only existed for unconjugated bilirubin. In addition, consistent findings existed when using maximum neonatal serum bilirubin as an exposure factor. Neonatal serum bilirubin levels are positively associated with childhood blood pressure/hypertension in preterm infants. Our findings may shed some light on the role of bilirubin in the prevention of hypertension.

## Introduction

Hypertension is one of the most serious public health burdens, and its prevalence has increased markedly worldwide [1, 2]. Hypertension-related mortality and morbidity are increasing, and high systolic blood pressure (SBP) is the largest contributor to global disability-adjusted life-years [2–4]. It has been demonstrated that blood pressure (BP) trajectories exist from childhood to adulthood, and children/young adults with higher BPs were prone to develop hypertension [5] and had a higher risk of cardiovascular disease (CVD) events as adults [6]. Therefore, elevated BP in children should be of significant concern, calling for early detection and intervention to prevent future cardiovascular complications.

Hypertension is a complex multifactor disorder, and combinations of genetic, environmental and social risk factors influence BP. According to the “developmental origins of health and disease” hypothesis [7], environmental exposures early in life can influence health status later in life. Some studies reported that childhood risk factors, including premature birth and exposure to some toxic substances, were associated with a higher risk of hypertension in later life [8, 9]. Bilirubin is an end product of heme catabolism in systemic circulation. Bilirubin can have toxic effects on developing neuronal tissues, and a high level of serum bilirubin is associated with neurological dysfunction in newborn babies [10, 11]. However, serum bilirubin is also a potent endogenous antioxidant and anti-inflammatory molecule under physiological conditions [12–14]. Numerous population-based studies have reported that serum bilirubin was negatively associated with SBP/hypertension [15, 16], ischemic heart disease [17], stroke [18] and CVD mortality [19–21]. One Mendelian randomization study demonstrated a strong association between higher bilirubin levels and a lower risk of CVD [22]. However, many other Mendelian randomization studies on this topic indicated that such associations were weak or even nonexistent [23–26]. To the best of our knowledge, the published studies on this topic were conducted in populations with normal or mildly elevated levels of serum bilirubin. During the neonatal period, the concentrations of serum bilirubin are commonly much higher than the concentrations in any other life stage. Does such a high level of serum bilirubin during the neonatal period have any associations with BP in later life? So far, no study has focused on this topic. In a previous study, researchers found that phototherapy for neonatal jaundice was positively associated with childhood asthma [27]. As a potential intermediate factor, phototherapy may alter any conclusions between neonatal jaundice and blood pressure. To exclude its influence, we used an old birth cohort dataset from a study in which participants were recruited before phototherapy was used routinely for the treatment of neonatal jaundice to investigate the correlations between neonatal serum bilirubin levels and childhood hypertension.

## Materials and methods

### Study population

The current study is a secondary data analysis. Data were obtained from the Collaborative Perinatal Project (CPP), and the data were publicly available through the U.S. National Archives (www.archives.gov/). Use of publicly available deidentified data does not require the approval of our Institutional Review Board.

The CPP was a multicenter birth cohort study in which 46,021 women with 56,990 pregnancies were recruited from 12 centers from 1959 to 1965 in the U.S. The offspring were followed up until 7 years of age. BP, height and other anthropometric parameters were measured and recorded by a trained observer using standardized procedures at each follow-up visit [28].

**Fig 1:**
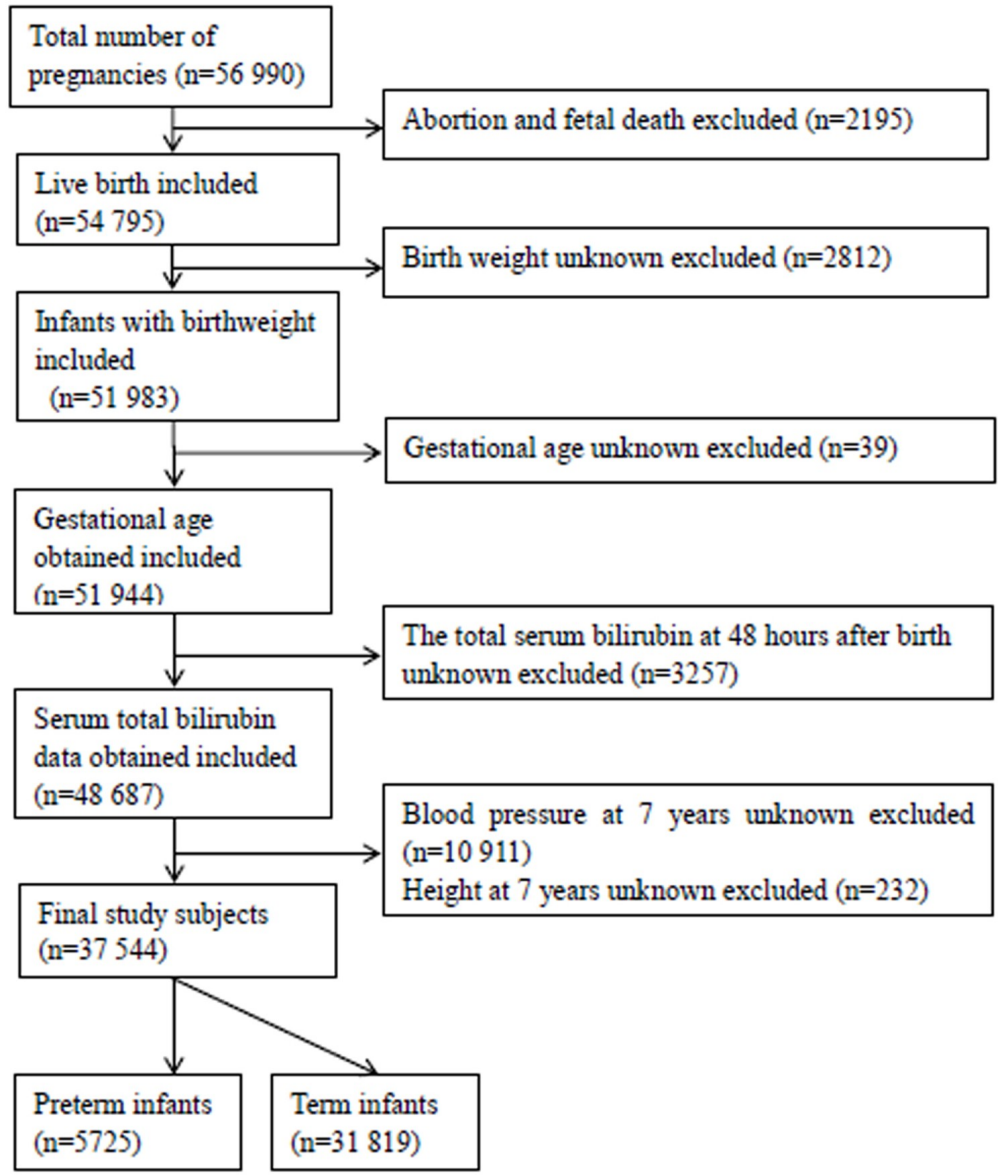
Flow chart of the selection of the study population from the U.S. Collaborative Perinatal Project birth cohort.

A total of 2,195 abortions or fetal deaths were initially excluded from this analysis. We excluded births with unknown birth weight (*n* = 2812) and unknown gestational age (*n* = 39). After excluding infants with an unknown neonatal total serum bilirubin (TSB) level 48 hours after birth (*n* = 3,257), subjects with an unknown BP (*n* = 10,911) or height (*n* = 232) at the age of 7 years were also excluded. Finally, the study included 37,544 infants: 31,819 term infants and 5,725 preterm infants (Fig 1).

### Bilirubin measurements

Bilirubin measurements were performed using the diazo method in 11 of the 12 centers and by direct spectrophotometry in the other centers. The coefficient of variation of standard specimens among the CPP laboratories was approximately 10%; the interlaboratory coefficient of variation was approximately 2% [29]. The study protocol of the CPP required the TSB levels of each newborn to be measured between 36 and 60 hours after birth, as close to 48 hours as possible. If the TSB level was greater than 10 mg/dl (171 μmol/L) in the first detection, a second test was performed 24 hours later. If the second TSB level exceeded 10 mg/dl, a third test was required to be performed when the infant was 4 to 5 days of age. The TSB level at 48 hours was used as the primary exposure, and the maximum TSB level was used to test the robustness of our findings. In several centers, conjugated bilirubin (CB) was routinely detected together with TSB. We also explored the associations between subtypes of bilirubin and childhood hypertension by stratifying the data by CB and unconjugated bilirubin (UCB).

### Outcomes and confounders

At the age of 7 years, prior to the physical examination and phlebotomy, BP measurement was performed once with a manual sphygmomanometer on the right arm of the child while they were in a seated position. Korotkoff phase 4 (muffling) or phase 5 (disappearance) was used for diastolic blood pressure (DBP) [30]. The distribution of BP was specifically calculated by height and sex. According to the fourth report on the diagnosis, evaluation, and treatment of hypertension in children and adolescents in 2004 [31], we defined hypertension as a SBP or DBP higher than the 90^th^ percentile for children of the same sex and height.

The following perinatal factors that influence both neonatal serum bilirubin and BP were chosen as potential confounders: maternal characteristics included hypertensive disorders during pregnancy (none, moderate, and severe), maternal smoking (0, 1–19 and 20 cigarette per day during pregnancy) and socioeconomic status (comprised of 5 categories as assessed by the original CPP investigators); newborn characteristics included race (white, black, and other race), sex (male and female), gestational age (as a categorical variable), birth weight (<2500 g, 2500–4000 g, and ≥4000 g) and infections during the neonatal period (with or without). A study indicated that transfusion in early life may influence the development of hypertension [32]. Thus, the performance of transfusion or exchange transfusion was also controlled in the statistical model.

### Statistical analysis

All statistical analyses were performed with SAS version 9.2 (SAS Institute, Cary, North Carolina) in the present study. Because the distribution of TSB was abnormal, the generalized linear model was used to adjust height at the age of 7 years and calculate its linear regression coefficient with BP at the age of 7 years in all study subjects. The pathophysiological mechanisms of neonatal jaundice and levels of serum bilirubin are different between term and preterm infants [33]. In the generalized linear model, we included gestational age (classified into preterm and term infants) and created a dummy variable to test the interaction between bilirubin and gestational age in relation to BP. The interaction existed, and we stratified the study subjects into term and preterm infants and performed further analyses.

In the next step, we categorized the babies into five groups based on levels of TSB: TSB < 3 mg/dl, 3 mg/dl ≤TSB< 6 mg/dl, 6 mg/dl ≤TSB<9 mg/dl, 9 mg/dl ≤TSB< 12 mg/dl, and TSB ≥ 12 mg/dl. The Cochran-Mantel-Haenszel Chi-square was used to estimate differences in the baseline characteristics among babies with different neonatal TSB concentrations. The associations between neonatal serum bilirubin and hypertension at the age 7 years were tested in the logistic model. A univariate logistic regression model (model 1) was used to calculate crude odds ratios (ORs) for hypertension at age 7 years in each group relative to the babies with neonatal TSB < 3 mg/dl. The adjusted ORs were calculated after adjusting the potential confounders in a multivariate logistic regression model (model 2). Because some women contributed data from more than one birth and children from the same family share similar genes and household environments, we used the generalized estimating equation model (model 3) to correct for intracluster correlations. In addition, we use the maximum TSB as the exposure to test the robustness of our findings.

## Results

Table 1 shows that among all infants included in this study, there were no association of neonatal TSB (per each 3 mg/dl) with SBP (0.23 mmHg; 95% CI: -0.01, 0.47, 174 mmHg) or DBP (-0.10 mmHg; 95% CI: -0.21, 0.02 mmHg) at the age of 7 years. Similar to TSB, no significant correlations of BP with either CB or UCB were identified. The correlations between neonatal bilirubin levels and BP were different in preterm and term babies (*P* <0.001), and interactions of gestational age and TSB with BP existed (*P* <0.005). Thus, Table 1 also shows the results of preterm and term infants. In preterm infants, a lower TSB and UCB levels of 3 mg/dl were associated with a higher systolic blood pressure of 0.62 mmHg (95% CI: 0.38–0.86 mmHg) and 0.70 mmHg (95% CI: 0.10–1.30 mmHg) respectively. However, in term infants, no such correlation was observed between neonatal serum bilirubin and SBP in our subjects. Furthermore, no significant correlation was observed between serum bilirubin and DBP in either preterm or term infants. Similar results were found when using maximum serum bilirubin values (S1 Table).

**Table 1:** The correlations of blood pressure with neonatal serum bilirubin 48 hours after birth, adjusted for height at 7 years old.

		Systolic Blood Pressure at 7 years old		Diastolic Blood Pressure at 7 years old	
		β (95% CI)	*P*	β (95% CI)	*P*
Total subjects	Total Serum Bilirubin	0.23 (-0.01, 0.47)	0.06	-0.10 (-0.21, 0.02)	0.08
	Conjugated Bilirubin	0.24 (-0.02, 0.50)	0.08	-0.08 (-0.33, 0.17)	0.51
	Unconjugated Bilirubin	0.00 (-0.11, 0.11)	0.99	0.03 (-0.08, 0.14)	0.55
Preterm infants	Total Serum Bilirubin	0.62 (0.38, 0.86)	<0.001	-0.03 (-0.27, 0.21)	0.79
	Conjugated Bilirubin	0.44 (-0.22, 1.10)	0.19	-0.16 (-0.49, 0.17)	0.62
	Unconjugated Bilirubin	0.70 (0.10, 1.30)	<0.05	-0.09 (-0.39, 0.21)	0.56
Term infants	Total Serum Bilirubin	-0.05 (-0.23, 0.08)	0.47	-0.13 (-0.27, 0.01)	0.06
	Conjugated Bilirubin	0.25 (-0.02, 0.52)	0.09	-0.07 (-0.33, 0.19)	0.58
	Unconjugated Bilirubin	-0.08 (-0.20, 0.04)	0.18	0.01 (-0.11, 0.13)	0.87

There was a significant difference in the incidence of hypertension between preterm and term infants (12.4% vs 11.2%, Chi-square = 5.15, *P* < 0.05). The baseline characteristics of the preterm and term babies with different levels of TSB concentrations are shown in S2 and S3 Tables.

Table 2 shows that in preterm infants, compared to the group with TSB levels < 3 mg/dl, there were positive associations of hypertension at the age of 7 years in infants with 3 mg/dl ≤ TSB <6 mg/dl (adjusted OR 1.36; 95% CI: 0.98, 1.89), 6 mg/dl ≤ TSB <9 mg/dl (adjusted OR 1.35; 95% CI: 0.98, 1.85), 9 mg/dl≤ TSB <12 mg/dl (adjusted OR 1.55; 95% CI: 1.10, 2.19), and ≥12 mg/dl (adjusted OR 1.42; 95% CI: 1.01, 2.00) (Table 2, Model 2). In term infants, compared to the group with TSB <3 mg/dl, there were no significant associations between bilirubin and hypertension at age 7 years in the other groups of infants. Using a generalized estimating equation model to correct for intracluster correlation (Table 2, Model 3) or using maximum serum bilirubin as an independent factor (S4 Table), similar results were found.

**Table 2:** The odds ratios of high blood pressure at age of 7 years with different concentrations of total serum bilirubin at 48h after birth.

	Total serum bilirubin	High blood pressure at 7 years old						
		n/N (%)	Model 1		Model 2		Model 3	
			OR	95% CI	OR	95% CI	OR	95% CI
Preterm Infants	< 3mg/dl	55/613 (9.0)	1		1		1	
	≥ 3mg/dl, < 6mg/dl	148/1270 (11.7)	1.34	0.97, 1.85	1.36	0.98, 1.89	1.36	0.98, 1.91
	≥ 6mg/dl, < 9mg/dl	202/1711 (11.8)	1.36	0.99, 1.86	1.35	0.98, 1.85	1.36	0.99, 1.86
	≥ 9mg/dl, < 12mg/dl	136/951 (14.3)	1.69	1.22, 2.36	1.55	1.10, 2.19	1.54	1.09, 2.21
	≥ 12mg/dl	169/1180 (14.3)	1.70	1.23, 2.34	1.42	1.01, 2.00	1.42	1.00, 2.02
Term Infants	< 3mg/dl	744/6452 (11.5)	1		1		1	
	≥ 3mg/dl, < 6mg/dl	1197/10510 (11.4)	0.99	0.90, 1.09	1.00	0.91, 1.11	1.01	0.91, 1.12
	≥ 6mg/dl, < 9mg/dl	1005/9248 (10.9)	0.94	0.85, 1.03	0.97	0.88, 1.08	0.96	0.85, 1.07
	≥ 9mg/dl, < 12mg/dl	392/3315 (11.8)	1.03	0.90, 1.17	1.07	0.93, 1.22	1.07	0.91, 1.25
	≥ 12mg/dl	226/2294 (11.6)	1.01	0.87, 1.17	1.02	0.88, 1.19	1.00	0.85, 1.24

Model 1: crude odds ratios; Model 2: Adjusted for race (white, black, and other race), sex (male and female), birth weight (< 2500g, 2500g–4000g and ≥4000g), gestational age (as a categorical variable), transfusion (yes and no), hypertensive disorders during pregnancy (none, moderate, and severe), maternal smoking (0, 1–19 and 20 cigarette per day during pregnancy) and socioeconomic status (comprised of 5 categories as assessed by the original CPP investigators); Model 3: adjusted for the same factors as model 2 in Generalized Estimating Equation mode

Table 3 shows that in preterm babies, the serum UCB and CB levels had different associations with hypertension at the age of 7 years. Consistent with the results of TSB, compared with the group with UCB <3 mg/dl, the adjusted ORs of hypertension were 1.54 (95% CI: 0.93, 2.55), 1.55 (95% CI: 0.99, 2.43), 1.51 (95%CI: 0.95, 2.41), and 1.54 (95% CI: 0.97, 2.44) among babies with 3 mg/dl≤ UCB <6 mg/dl, 6 mg/dl≤ UCB <9 mg/dl, 9 mg/dl≤ UCB <12 mg/dl and UCB ≥12 mg/dl, respectively. However, compared with babies with serum CB <1 mg/dl, the adjusted ORs of hypertension at the age of 7 years were 1.11 (95% CI: 0.80, 1.53) and 0.85 (95% CI: 0.48, 1.50) among babies with 1 mg/dl≤ CB <2 mg/dl and CB ≥2 mg/dl, respectively. After correcting for the intracluster correlation in model 3, the above results were similar. In term babies, two subtypes of neonatal bilirubin had no significant associations with childhood hypertension (S5 Table).

**Table 3:** The odds ratios of high blood pressure at the age of 7 years in preterm infants with different concentrations and subtypes of serum bilirubin at 48h after birth.

Bilirubin		High blood pressure at the age of 7 years						
		n/N (%)	Model 1		Model 2		Model 3	
			OR	95% CI	OR	95% CI	OR	95% CI
Unconjugated bilirubin ^a^	< 3mg/dl	29/317 (9.2)	1	-	1	-	1	-
	≥ 3mg/dl, < 6mg/dl	43/326 (13.2)	1.51	0.92, 2.49	1.54	0.93, 2.55	1.55	0.93, 2.57
	≥ 6mg/dl, <9mg/dl	89/659 (13.5)	1.61	1.04, 2.50	1.55	0.99, 2.43	1.55	1.00, 2.46
	≥ 9mg/dl, < 12mg/dl	75/539 (13.9)	1.63	1.04, 2.56	1.51	0.95, 2.41	1.51	0.95, 2.42
	≥ 12mg/dl	109/728 (15.0)	1.79	1.16, 2.75	1.54	0.97, 2.44	1.54	0.96, 2.46
Conjugated bilirubin^b^	< 1mg/dl	272/2074 (13.1)	1	-	1	-	1	-
	≥ 1mg/dl, < 2mg/dl	58/378 (15.4)	1.20	0.88, 1.63	1.11	0.80, 1.53	1.08	0.73, 1.53
	≥ 2mg/dl	15/117 (12.8)	0.95	0.55, 1.66	0.85	0.48, 1.50	0.88	0.47, 1.58

Model 1: crude odds ratios; Model 2: Adjusted for race (white, black, and other races), sex (male and female), gestational age (as a categorical variable), birth weight (<2500g, and ≥2500g), transfusion (yes and no), hypertensive disorders during pregnancy (none, moderate, and severe), maternal smoking (0, 1–19 and 20 cigarette per day during pregnancy) and socioeconomic status (comprised of 5 categories as assessed by the original CPP investigators); Model 3: adjusted for the same factors as model 2 in Generalized Estimating Equation model

^a^ Adjusted by direct serum bilirubin additionally;

^b^ Adjusted by indirect serum bilirubin additionally;

## Discussion

In the present study, we first reported positive associations between neonatal bilirubin and SBP/hypertension at the age of 7 years in preterm infants. The findings also indicated that a lower TSB and UCB levels of 3 mg/dl were associated with a higher blood pressure of 62 mmHg and 0.70 mmHg, respectively. In full-term infants, no subtypes of serum bilirubin were correlated with BP or hypertension at the age of 7 years.

Neonatal jaundice is a condition commonly encountered in infants, and most cases are physiological. However, due to its potential neurotoxicity, the prophylactic use of phototherapy for neonatal jaundice, especially in preterm babies, is widely employed in current clinical practice [34]. A previous study found that phototherapy for neonatal jaundice was a risk factor for childhood asthma [27]. In the study on the present topic, phototherapy is a potential intermediate factor and may alter any conclusions between neonatal jaundice and blood pressure. Thus, by using current data, it is difficult to avoid the effect of phototherapy and to clarify the association between neonatal jaundice and childhood hypertension. In the CPP study period (1959–1965), phototherapy was not yet routinely used for neonatal jaundice, and this is the only population dataset with different levels of bilirubin before phototherapy was widely used. These conditions made the CPP data particularly suitable to study the exposure-effect relationships of neonatal bilirubin and childhood BP/hypertension.

BP is influenced by multiple factors, and hypertension is partly characterized by low-grade inflammatory symptoms [35, 36]. Previous studies have reported that bilirubin has anti-inflammatory and antioxidant effects [12–14]. Most studies demonstrated that high bilirubin levels were associated with a lower risk of hypertension, stroke, IHD, and other CVDs [15–22]. Some researchers have suggested that bilirubin could be a possible substance to be used for cardiometabolic disorder interventions [37, 38]. Although the possible protective effects of bilirubin have been shown in some studies, inconsistent results still exist [23–26]. Furthermore, bilirubin also has well-known toxic effects on the nervous system, especially on developing nervous tissue in preterm infants [39]. Due to the insufficiency of hepatic uptake and conjugation and the hyperfunction of enterohepatic circulation, preterm infants are more likely to suffer from hyperbilirubinemia than term babies [40]. Despite the wide prophylactic use of phototherapy for neonatal jaundice in current clinical practice, there is a lack of evidence from long-term cardiovascular outcomes to determine the appropriate therapeutic strategy for neonatal jaundice. By using prospective data from the U.S. CPP, the present study first found that bilirubin in the neonatal period was positively associated with childhood SBP/hypertension in preterm infants. However, in term babies, no association of neonatal bilirubin with childhood hypertension was observed in the current study. These findings might somehow lend some credence to the strategy of intervening in newborn cases of hyperbilirubinemia as early as possible in future clinical practice.

The pathogenesis of hypertension involves an increase in sympathetic nervous system activity and renin-angiotensin-aldosterone system activity [41]. Neurological disorders can affect the stabilization of the autonomic nervous system. Bilirubin is deposited in the globus pallidus, hypothalamus and brain stem in children with jaundice [42]. The paraventricular nucleus of the hypothalamus plays a key role in cardiovascular activity. We speculate that the excitability of sympathetic nerve fibers, which is positively associated with the bilirubin deposited in the central nervous system, is an important factor in the pathogenesis of hypertension. The findings of our analysis revealed that the positive correlations between bilirubin levels and childhood hypertension were only present in preterm babies. It can be considered that the blood-brain barrier of premature infants is less developed than that of full-term infants and that bilirubin can easily diffuse into the central nervous system. We also found that the positive correlations were more significant with UCB than with CB. This can be plausibly interpreted by the chemical properties of the two kinds of bilirubin. UCB is a lipid-soluble molecule that easily combines with phospholipid-rich neurons and forms precipitates, resulting in brain injury [43]. In contrast, CB is a water-soluble molecule that does not undergo the abovementioned process.

### Strengths and limitations

Our study has several limitations, as it is a historic study performed using data collected 50 years ago. For active interventions for neonatal jaundice, especially in preterm infants in current clinical practice, the relevance of our findings in contemporary populations may be questioned. However, for the same reason, our data may have provided the only opportunity to disclose the correlations between bilirubin levels and childhood hypertension in natural conditions in preterm infants. Furthermore, there is no agreement regarding phototherapy for preterm infants in clinical practice. Although prophylactic phototherapy has been proven to be beneficial for preterm infants, the related evidence is still limited [44]. Our findings may add some insights on this topic of preventing the development of childhood hypertension. Second, even though the CPP was carefully conducted with a high long-term follow-up rate (mean = 88%) [45], the selection bias from the loss of BP at 7 years of age and some unknown confounders cannot be avoided. Furthermore, because it was based on an observational study, the present analysis was unable to determine whether elevated bilirubin is the causal factor of BP elevation, and more interventional studies should be conducted to determine the real relationship.

## Conclusions

Our study was the first to investigate the association between neonatal serum bilirubin and BP at the age of 7 years. There was no significant association between serum bilirubin and childhood BP in the whole study populations of in the subgroup of term infants. Interestingly, a positive association was observed between serum bilirubin and BP/hypertension in preterm infants. This positive association only existed for UCB. Currently, the incidence rates of premature birth and hypertension are increasing, and our findings may shed some light on the role of bilirubin in the prevention and treatment of hypertension.

## Supporting information

S1 Table The correlations of blood pressure with neonatal maximum serum bilirubin after birth, adjusted for height at age of 7 years. (DOCX)

S2 Table Baseline characteristics of preterm infants with different concentrations of total serum bilirubin. (DOCX)

S3 Table Baseline characteristics of term infants with different concentrations of total serum bilirubin. (DOCX)

S4 Table The odds ratios of high blood pressure at the age of 7 years with different concentrations of neonatal maximum total serum bilirubin. (DOCX)

S5 Table The odds ratios of high blood pressure at age of 7 years in term newborns with different concentrations and subtypes of serum bilirubin at 48h after birth. (DOCX)

S1 Fig The reason for the stratification of gestational age. (PDF)
