# Supplementary material for: Associations between neonatal serum bilirubin and childhood hypertension
Source: PLoS One. 2019 Jul 18;14(7):e0219942. doi: 10.1371/journal.pone.0219942 (PMC6638957; doi:10.1371/journal.pone.0219942)
Supplement: S1 Table — (DOCX) [file pone.0219942.s001.docx]

S1 Table: The Correlations of Blood Pressure with Neonatal Maximum Serum Bilirubin After Birth, adjusted for Height at Age of 7 Years.

|  | | Systolic Blood Pressure at the age of 7 years | | Diastolic Blood Pressure at the age of 7 years | |
| --- | --- | --- | --- | --- | --- |
|  |  | β (95% CI) | *P* | β (95% CI) | *P* |
| All subjects | Total Serum Bilirubin | -0.02 (-0.12, 0.08) | 0.71 | -0.19 (-0.39, 0.01) | 0.05 |
|  | Conjugated Bilirubin | 0.28 (-0.19, 0.15) | 0.33 | -0.14 (-0.41, 0.13) | 0.31 |
|  | Unconjugated Bilirubin | -0.03 (-0.15, 0.09) | 0.57 | 0.07 (-0.05, 0.19) | 0.21 |
| Preterm infants | Total Serum Bilirubin | 0.78 (0.53, 1.03) | <0.0001 | -0.02 (-0.27, 0.23) | 0.86 |
|  | Conjugated Bilirubin | 0.33 (0.00, 0.66) | <0.05 | 0.07 (-0.76, 0.90) | 0.87 |
|  | Unconjugated Bilirubin | 0.90 (0.06, 1.74) | <0.05 | 0.11 (-0.21, 0.43) | 0.52 |
| Term infants | Total Serum Bilirubin | -0.09 (-0.20, 0.02) | 0.10 | -0.21 (-0.51, 0.09) | 0.23 |
|  | Conjugated Bilirubin | 0.16 (-0.12, 0.44) | 0.38 | -0.18 (-0.44, 0.12) | 0.26 |
|  | Unconjugated Bilirubin | -0.11 (-0.24, 0.02) | 0.09 | 0.04 (-0.08, 0.16) | 0.55 |
