# Supplementary material for: Associations between neonatal serum bilirubin and childhood hypertension
Source: PLoS One. 2019 Jul 18;14(7):e0219942. doi: 10.1371/journal.pone.0219942 (PMC6638957; doi:10.1371/journal.pone.0219942)
Supplement: S2 Table — (DOCX) [file pone.0219942.s002.docx]

S2 Table: Baseline Characteristics of Preterm Infants with Different Concentrations of Total Serum Bilirubin.

| Total Serum Bilirubin | | < 3mg/dl  (N=613) | ≥3mg/dl, < 6mg/dl (N=1270) | | ≥6mg/dl, <9mg/dl (N=1711) | ≥9mg/dl, < 12mg/dl (N=951) | ≥12mg/dl  (N=1180) | P |
| --- | --- | --- | --- | --- | --- | --- | --- | --- |
| Male; N (%) | 252 (41.2) | | | 644 (50.7) | 879 (51.4) | 487 (51.2) | 672 (57.0) | <0.001 |
| Race; N (%) | |  |  | |  |  |  |  |
| White | | 467 (76.1) | 974 (76.7) | | 1216 (71.1) | 607 (63.8) | 624 (52.9) | <0.001 |
| Black | | 107 (17.5) | 237 (18.7) | | 407 (23.8) | 307 (32.3) | 515 (43.6) |  |
| Others | | 39 (6.4) | 59 (4.7) | | 88 (5.1) | 37 (3.9) | 41 (3.5) |  |
| Brithweight; N (%) | |  |  | |  |  |  | <0.0001 |
| <2500g | | 81 (13.2) | 208 (16.4) | | 443 (25.9) | 421 (44.3) | 671 (56.9) |  |
| ≥ 2500g | | 532 (86.8) | 1062 (83.6) | | 1268 (74.1) | 530 (55.7) | 509 (43.1) |  |
| Gestational age; N (%) | | |  | |  |  |  | < 0.001 |
| ≤ 28W | | 58 (9.5) | 88 (6.9) | | 119 (6.9) | 67 (7.0) | 89 (7.6) |  |
| 28^+1^ - 32W | | 104 (17.0) | 204 (16.1) | | 270 (15.8) | 166 (17.5) | 260 (22.0) |  |
| 32^+1^ - 34W | | 151 (24.6) | 298 (23.5) | | 410 (24.0) | 226 (23.8) | 307 (26.0) |  |
| 34^+1^ – 36^+6^W | | 300 (48.9) | 680 (53.5) | | 912 (53.3) | 492 (51.7) | 524 (44.4) |  |
| Hypertensive disorders during pregnancy; N (%) | | | | |  |  |  | < 0.0001 |
| None | | 380 (62.0) | 865 (68.1) | | 1303 (76.1) | 729 (76.7) | 888 (75.3) |  |
| Moderate | | 197(30.5) | 358 (28.2) | | 332 (19.4) | 192 (20.2) | 240 (20.3) |  |
| Severe | | 41 (6.7) | 42 (3.3) | | 70 (4.1) | 24 (2.5) | 37 (3.1) |  |
| Unknown | | 5 (0.8) | 5 (0.5) | | 6 (0.4) | 6 (0.6) | 15 (1.3) |  |
| Maternal age; N (%) | | |  | |  |  |  | < 0.05 |
| < 20 | | 180 (29.4) | 396 (31.2) | | 527 (30.8) | 281 (29.6) | 296 (25.1) |  |
| 20 - 34 | | 363 (59.2) | 787 (62.0) | | 1055 (61.7) | 594 (62.5) | 784 (66.4) |  |
| ≥ 35 | | 70 (11.4) | 87 (6.9) | | 129 (7.5) | 76 (8.0) | 100 (8.5) |  |
| Maternal smoking; N (%) | | |  | |  |  |  | < 0.005 |
| 0 | | 309 (50.4) | 646 (50.9) | | 922 (53.9) | 466 (49.0) | 559 (47.4) |  |
| 1-19 | | 215 (35.1) | 446 (35.1) | | 542 (31.7) | 301 (31.7) | 379 (32.1) |  |
| ≥ 20 | | 76 (12.4) | 146 (11.5) | | 204 (11.9) | 153 (16.1) | 209 (17.7) |  |
| Unknown | | 13 (2.1) | 32 (2.5) | | 43 (2.5) | 31 (3.3) | 33 (2.8) |  |
| Socioeconomic status; N (%) | | |  | |  |  |  | <0.001 |
| 1 | | 87 (14.2) | 182 (14.3) | | 219 (12.8) | 94 (9.9) | 94 (8.0) |  |
| 2 | | 233 (38.0) | 462 (36.4) | | 659 (38.5) | 359 (37.8) | 336 (28.5) |  |
| 3 | | 183 (29.9) | 381 (30.0) | | 502 (29.3) | 303 (31.9) | 371 (31.4) |  |
| 4 | | 72 (11.8) | 161 (12.7) | | 219 (12.8) | 109 (11.5) | 219 (18.6) |  |
| 5 | | 15 (2.5) | 42 (3.3) | | 67 (3.9) | 65 (6.8) | 108 (9.2) |  |
| Unknown | | 23 (3.8) | 42 (3.3) | | 45 (2.6) | 21 (2.2) | 52 (4.4) |  |
