# Supplementary material for: Associations between neonatal serum bilirubin and childhood hypertension
Source: PLoS One. 2019 Jul 18;14(7):e0219942. doi: 10.1371/journal.pone.0219942 (PMC6638957; doi:10.1371/journal.pone.0219942)
Supplement: S3 Table — (DOCX) [file pone.0219942.s003.docx]

S3 Table: Baseline Characteristics of Term Infants with Different Concentrations of Total Serum Bilirubin.

| Total Serum Bilirubin | | < 3mg/dl  (N=6452) | ≥3mg/dl, < 6mg/dl (N=10 510) | ≥6mg/dl, <9mg/dl (N=9248) | ≥9mg/dl, < 12mg/dl (N=3315) | ≥12mg/dl  (N=2294) | P |
| --- | --- | --- | --- | --- | --- | --- | --- |
| Male; N (%) | 2911 (45.1) | | 5075 (48.3) | 4923 (53.2) | 1814 (54.7) | 1361 (59.3) | <0.001 |
| Race; N (%) | |  |  |  |  |  | <0.001 |
| White | | 3384 (52.5) | 5346 (50.9) | 4497 (48.6) | 1617 (48.8) | 1241 (54.1) |  |
| Black | | 2874 (44.5) | 4833 (46.0) | 4396 (47.5) | 1562 (47.1) | 948 (41.3) |  |
| Others | | 194 (3.0) | 331 (3.2) | 355 (3.9) | 136 (4.1) | 105 (4.6) |  |
| Brithweight; N (%) | |  |  |  |  |  | <0.0001 |
| <2500g | | 265 (4.1) | 446 (4.2) | 497 (5.4) | 226 (6.8) | 249 (10.9) |  |
| ≥ 2500g, <4000g | | 5686 (88.1) | 9390 (89.3) | 8343 (90.2) | 2916 (88.0) | 1911 (83.3) |  |
| ≥ 4000g | | 501 (7.8) | 674 (6.4) | 408 (4.4) | 173 (5.2) | 134 (5.8) |  |
| Gestational age; N (%) | | |  |  |  |  | < 0.0001 |
| 37 - 37^+6^W | | 318 (4.9) | 683 (6.5) | 834 (9.0) | 370 (11.2) | 379 (16.5) |  |
| 38 - 38^+6^W | | 671 (10.4) | 1439 (13.7) | 1596 (17.3) | 589 (17.8) | 422 (18.4) |  |
| 39 - 39^+6^W | | 1286 (19.9) | 2266 (21.6) | 2223 (24.0) | 751 (22.7) | 513 (22.4) |  |
| 40 - 40^+6^W | | 1623 (25.2) | 2528 (24.1) | 2059 (22.3) | 713 (21.5) | 442 (19.3) |  |
| 41 - 41^+6^W | | 1234 (19.1) | 1738 (16.5) | 1242 (13.4) | 399 (12.0) | 261 (11.4) |  |
| ≥ 42W | | 1320 (20.5) | 1856 (17.7) | 1294 (14.0) | 493 (14.9) | 277 (12.1) |  |
| Hypertensive disorders during pregnancy; N (%) | | | |  |  |  | < 0.001 |
| None | | 4388 (68.0) | 7505 (71.4) | 6735 (72.8) | 2450 (73.9) | 1685 (73.5) |  |
| Moderate | | 1852 (28.7) | 2682 (25.5) | 2277 (24.6) | 760 (22.9) | 542 (23.6) |  |
| Severe | | 187 (2.9) | 297 (2.8) | 209 (2.3) | 97 (2.9) | 62 (2.7) |  |
| Unknown | | 25 (0.4) | 26 (0.3) | 27 (0.3) | 8 (0.2) | 5 (0.2) |  |
| Maternal age; N (%) | | |  |  |  |  | < 0.005 |
| < 20 | | 1287 (20.0) | 2295 (21.8) | 2225 (24.1) | 826 (24.9) | 481 (21.0) |  |
| 20 - 34 | | 4544 (70.4) | 7367 (70.1) | 6384 (69.0) | 2251 (67.9) | 1598 (69.7) |  |
| ≥ 35 | | 621 (9.8) | 848 (8.1) | 639 (6.9) | 238 (7.2) | 215 (9.4) |  |
| Maternal smoking; N (%) | | |  |  |  |  | < 0.005 |
| 0 | | 2987 (46.3) | 5412 (51.5) | 5218 (56.4) | 1940 (58.5) | 1354 (59.0) |  |
| 1-19 | | 2026 (31.4) | 3258 (31.0) | 2600 (28.1) | 919 (27.7) | 581 (25.3) |  |
| ≥ 20 | | 1288 (20.0) | 1590 (15.1) | 1811 (12.8) | 370 (11.2) | 296 (12.9) |  |
| Unknown | | 151 (2.3) | 250 (2.4) | 249 (2.7) | 86 (2.6) | 63 (2.8) |  |
| Socioeconomic status; N (%) | | |  |  |  |  | <0.05 |
| 1 | | 441 (6.8) | 724 (6.9) | 729 (7.9) | 228 (6.9) | 158 (6.9) |  |
| 2 | | 1748 (27.1) | 3069 (29.2) | 2708 (29.3) | 1063 (32.1) | 628 (27.4) |  |
| 3 | | 2024 (31.4) | 3201 (30.5) | 2763 (29.9) | 975 (29.4) | 704 (30.7) |  |
| 4 | | 1403 (21.8) | 2204 (21.0) | 1828 (19.8) | 626 (18.9) | 477 (20.8) |  |
| 5 | | 680 (10.5) | 1107 (10.5) | 1029 (11.1) | 343 (10.4) | 264 (11.5) |  |
| Unknown | | 156 (2.4) | 205 (2.0) | 191 (2.1) | 80 (2.4) | 63 (2.8) |  |
