# Supplementary material for: Associations between neonatal serum bilirubin and childhood hypertension
Source: PLoS One. 2019 Jul 18;14(7):e0219942. doi: 10.1371/journal.pone.0219942 (PMC6638957; doi:10.1371/journal.pone.0219942)
Supplement: S4 Table — (DOCX) [file pone.0219942.s004.docx]

S4 Table: The Odds Ratios of High Blood Pressure at the Age of 7 Years with Different Concentrations of Neonatal Maximum Total Serum Bilirubin.

|  | Total serum bilirubin | High blood pressure at the age of 7 years | | | | | | |
| --- | --- | --- | --- | --- | --- | --- | --- | --- |
|  |  |  | Model 1 | | Model 2 | | Model 3 | |
|  |  | n (%) | OR | 95% CI | OR | 95% CI | OR | 95% CI |
| Preterm | < 3mg/dl | 59/634 (9.3) | 1 |  | 1 |  | 1 |  |
|  | ≥ 3mg/dl, < 6mg/dl | 172/1414 (12.2) | 1.35 | 0.99, 1.84 | 1.36 | 0.99, 1.86 | 1.35 | 0.98, 1.88 |
|  | ≥ 6mg/dl, < 9mg/dl | 254/2163 (11.7) | 1.30 | 0.96, 1.75 | 1.26 | 0.92, 1.70 | 1.27 | 0.92, 1.73 |
|  | ≥ 9mg/dl, < 12mg/dl | 124/872 (14.2) | 1.62 | 1.16, 2.24 | 1.49 | 1.06, 2.09 | 1.49 | 1.05, 2.12 |
|  | ≥ 12mg/dl | 101/642 (15.7) | 1.82 | 1.29, 2.56 | 1.51 | 1.06, 2.17 | 1.53 | 1.05, 2.22 |
| Term | < 3mg/dl | 774/6636 (11.7) | 1 |  | 1 |  | 1 |  |
|  | ≥ 3mg/dl, < 6mg/dl | 1221/10713 (11.4) | 0.97 | 0.89, 1.07 | 0.99 | 0.90, 1.09 | 1.00 | 0.90, 1.11 |
|  | ≥ 6mg/dl, < 9mg/dl | 1044/9618 (10.9) | 0.92 | 0.84, 1.02 | 0.95 | 0.86, 1.05 | 0.95 | 0.86, 1.07 |
|  | ≥ 9mg/dl, < 12mg/dl | 438/3708 (11.8) | 1. 01 | 0.90, 1.15 | 1.04 | 0.91, 1.20 | 1.03 | 0.90, 1.21 |
|  | ≥ 12mg/dl | 127/1143 (11.1) | 0.99 | 0.84, 1.16 | 0.99 | 0.84, 1.17 | 0.99 | 0.83, 1.20 |

Model 1: crude odds ratios;

Model 2: Adjusted for race (white, black, and other races), sex (male and female), gestational age (as a categorical variable), transfusion (yes and no), hypertensive disorders during pregnancy (none, moderate, and severe), maternal smoking (0, 1-19 and 20 cigarette per day during pregnancy ) and socioeconomic status (comprised of 5 categories as assessed by the original CPP investigators); ^a^ Adjusted for birth weight (<2500g and ≥2500g); ^b^ Adjusted for birth weight (< 2500g, 2500g - 4000g and ≥4000g);

Model 3: adjusted for the same factors as model 2 in Generalized Estimating Equation model.
