# Supplementary material for: Associations between neonatal serum bilirubin and childhood hypertension
Source: PLoS One. 2019 Jul 18;14(7):e0219942. doi: 10.1371/journal.pone.0219942 (PMC6638957; doi:10.1371/journal.pone.0219942)
Supplement: S5 Table — (DOCX) [file pone.0219942.s005.docx]

S5 Table: The Odds Ratios of High Blood Pressure at Age of 7 Years in Term Newborns with Different Concentrations and Subtypes of Serum Bilirubin at 48h After Birth.

| Bilirubin | | High blood pressure at 7 years old | | | | | | | | |
| --- | --- | --- | --- | --- | --- | --- | --- | --- | --- | --- |
|  |  | Model 1 | | | Model 2 | | | Model 3 | | |
|  |  | n (%) | OR | 95%CI | | OR | 95%CI | | OR | 95%CI |
| Unconjugated bilirubin ^a^ | < 3mg/dl | 430/3824 (11.2) | 1 | - | | 1 | - | | 1 | - |
|  | ≥3mg/dl, < 6mg/dl | 374/3438 (10.9) | 0.96 | 0.83, 1.12 | | 0.97 | 0.84, 1.13 | | 0.97 | 0.82, 1.15 |
|  | ≥6mg/dl, <9mg/dl | 530/5036 (10.5) | 0.93 | 0.81, 1.06 | | 0.96 | 0.84, 1.10 | | 0.96 | 0.84, 1.11 |
|  | ≥9mg/dl, < 12mg/dl | 287/2651 (10.8) | 0.96 | 0.82, 1.12 | | 0.99 | 0.84, 1.17 | | 0.98 | 0.83, 1.19 |
|  | ≥12mg/dl | 206/1695 (12.2) | 1.09 | 0.92, 1.30 | | 1.13 | 0.94, 1.35 | | 1.13 | 0.94, 1.37 |
| Conjugated bilirubin ^b^ | < 1mg/dl | 1473/13927 (10.6) | 1 | - | | 1 | - | | 1 | - |
|  | ≥1mg/dl, <2mg/dl | 225/1711 (13.2) | 1.28 | 1.10, 1.49 | | 1.17 | 0.997, 1.36 | | 1.16 | 0.99, 1.38 |
|  | ≥ 2mg/dl | 129/1006 (12.8) | 1.24 | 1.03, 1.51 | | 1.03 | 0.84, 1.26 | | 1.03 | 0.84, 1.28 |

Model 1: crude odds ratios;

Model 2: Adjusted for race (white, black, and other races), sex (male and female), gestational age (as a categorical variable), birth weight (<2500g, 2500g-4000g, and ≥4000g), transfusion (yes and no), hypertensive disorders during pregnancy (none, moderate, and severe), maternal smoking (0, 1-19 and 20 cigarette per day during pregnancy ) and socioeconomic status (comprised of 5 categories as assessed by the original CPP investigators); ^a^ Adjusted by direct serum bilirubin additionally; ^b^Adjusted by indirect serum bilirubin additionally;

Model 3: adjusted for the same factors as model 2 in Generalized Estimating Equation model.
