# Supplementary material for: Associations between neonatal serum bilirubin and childhood hypertension
Source: PLoS One. 2019 Jul 18;14(7):e0219942. doi: 10.1371/journal.pone.0219942 (PMC6638957; doi:10.1371/journal.pone.0219942)
Supplement: S1 Fig — (PDF) [file pone.0219942.s006.pdf]

Fig 1. The reason for the stratification of gestational age.

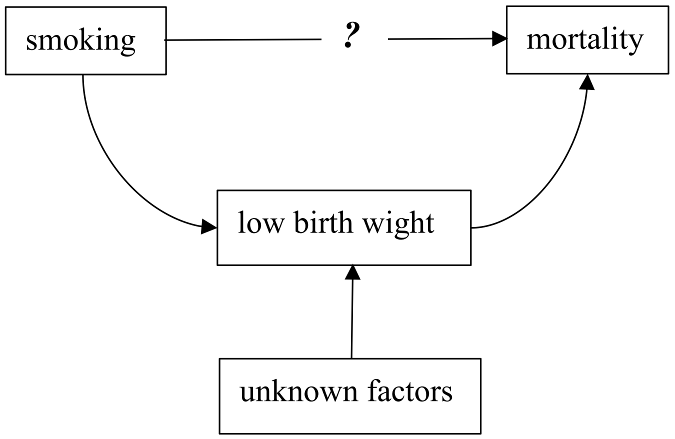

Fig A. The graph proposed low birth weight in the association between smoking and mortality.

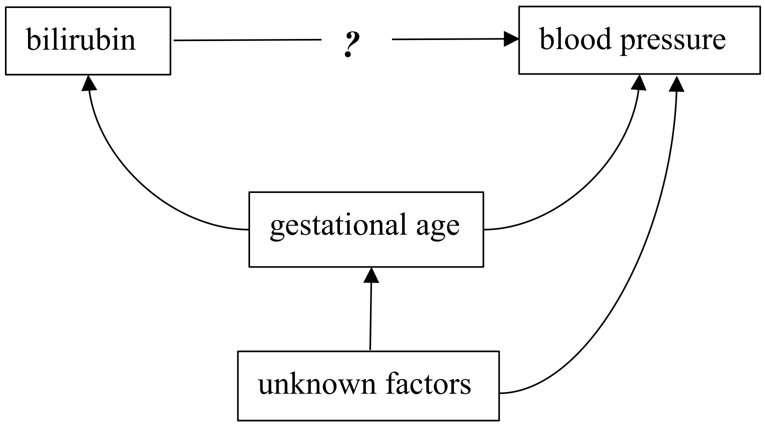

Fig B. The casual graph proposed gestational age in the association between bilirubin and blood pressure.
